# External validation of deep learning-derived 18F-FDG PET/CT delta biomarkers for loco-regional control in head and neck cancer

**DOI:** 10.2340/1651-226X.2025.43977

**Published:** 2025-08-30

**Authors:** David Gergely Kovacs, Marianne Aznar, Marcel van Herk, Iskandar Mohamed, James Price, Claes Nøhr Ladefoged, Barbara Malene Fischer, Flemming Littrup Andersen, Andrew McPartlin, Eliana M. Vasquez Osorio, Azadeh Abravan

**Affiliations:** a Department of Clinical Physiology and Nuclear Medicine, Rigshospitalet, Copenhagen, Denmark; b Department of Clinical Medicine, University of Copenhagen, Copenhagen, Denmark; c Division of Cancer Sciences, University of Manchester, Manchester, the United Kingdom of Great Britain and Northern Ireland; d The Christie NHS Foundation Trust, Manchester, the United Kingdom of Great Britain and Northern Ireland; e Department of Applied Mathematics and Computer Science, Technical University of Denmark, Lyngby, Denmark; f Princess Margaret Cancer Centre, University of Toronto, Toronto, Canada

**Keywords:** Head and neck neoplasms, positron emission tomography computed tomography, neural networks (computer), radiotherapy, biomarkers

## Abstract

**Background and purpose:**

Delta biomarkers that reflect changes in tumour burden over time can support personalised follow-up in head and neck cancer. However, their clinical use can be limited by the need for manual image segmentation. This study externally evaluates a deep learning model for automatic determination of volume change from serial 18F-fluorodeoxyglucose (18F-FDG) positron emission tomography/computed tomography (PET/CT) scans to stratify patients by loco-regional outcome.

**Patient/material and methods:**

An externally developed deep learning algorithm for tumour segmentation was applied to pre- and post-radiotherapy (RT, with or without concomitant chemoradiotherapy) PET/CT scans of 50 consecutive head and neck cancer patients from The Christie NHS Foundation Trust, UK. The model, originally trained on pre-treatment scans from a different institution, was deployed to derive tumour volumes at both time points. The AI-derived change in tumour volume (ΔPET-Gross tumour volume (GTV)) was calculated for each patient. Kaplan-Meier analysis assessed loco-regional control based on ΔPET-GTV, dichotomised at the cohort median. In a separate secondary analysis confined to the pre‑treatment scans, a radiation oncologist qualitatively evaluated the AI‑generated PET‑GTV contours.

**Results:**

Patients with higher ΔPET-GTV (i.e. greater tumour shrinkage) had significantly improved loco-regional control (log-rank p = 0.02). At 2 years, control was 94.1% (95% CI: 83.6–100%) vs. 53.6% (95% CI: 32.2–89.1%). Only one of nine failures occurred in the high ΔPET-GTV group. Clinician review found AI volumes acceptable for planning in 78% of cases. In two cases, the algorithm identified oropharyngeal primaries on pre-treatment PET-CT before clinical identification.

**Interpretation:**

Deep learning-derived ΔPET-GTV may support clinically meaningful assessment of post-treatment disease status and risk stratification, offering a scalable alternative to manual segmentation in PET/CT follow-up.

## Graphical abstract

**Figure:**
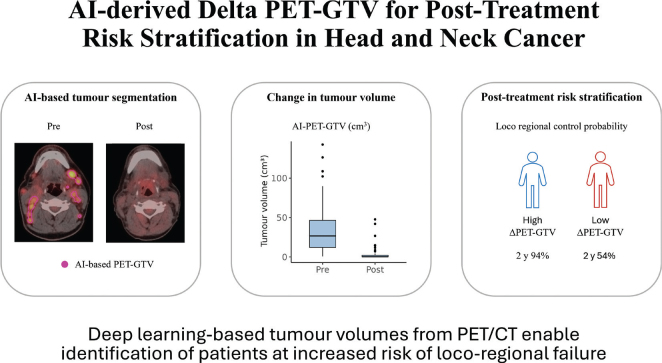

## Introduction

Head and neck cancer (HNC) is a heterogeneous group of malignancies with complex treatment pathways and variable outcomes [1]. Despite advances in radiotherapy (RT), imaging and systemic therapy, loco-regional recurrence remains a major cause of morbidity and mortality, particularly in patients with advanced-stage disease [2–4]. The burden of disease is further compounded by marked sociodemographic disparities. Risk factors such as tobacco and alcohol use, poor oral health and limited access to preventive care are more prevalent in lower socioeconomic groups, contributing to both incidence and worse outcomes [5]. Current follow-up strategies after treatment often rely on fixed schedules of clinical examination and imaging and are not adjusted to individual risk. As a result, both under- and over-surveillance are common, with implications for healthcare resources and timely detection of recurrence, with under-surveillance potentially limiting opportunities for salvage treatment [6, 7]. There is a growing recognition of the need for personalised follow-up protocols based on prognostic biomarkers that can stratify patients by risk and guide post-treatment care [8].

Imaging biomarkers derived from 18F-fluorodeoxyglucose positron emission tomography/computed tomography (18F-FDG PET/CT) are amongst the most studied in HNC [9–11]. Baseline FDG uptake has been linked to tumour aggressiveness and prognosis [4], and response evaluation after chemoradiotherapy using PET/CT is widely implemented [12, 13]. However, the clinical application of ‘delta biomarkers’ (quantitative measures of change between baseline and follow-up imaging) has been limited by the need for manual image segmentation, which is time-consuming and prone to interobserver variability [10, 14].

Recent advances in deep learning have enabled automated, scalable and standardised detection, segmentation and extraction of imaging biomarkers across modalities and clinical domains [15–17]. Frameworks such as nnU-Net have demonstrated robust, self-configuring segmentation performance across biomedical datasets without manual tuning [15]. In HNC, deep learning models trained on PET/CT have shown high concordance with expert-defined tumour volumes and promising generalisability across institutions [18–21]. This includes studies evaluating the contribution of different imaging modalities [18] and benchmarking efforts such as the HECKTOR challenge, which have accelerated development and external comparison [20]. Accurate tumour segmentation forms the basis for extracting volumetric biomarkers from PET/CT, such as baseline tumour burden or changes over time. These imaging-derived metrics can then be integrated into prognostic models to estimate individualised risk of recurrence or treatment failure [20–22].

Although studies in other tumour types suggest that changes in tumour volume may carry prognostic value, there is limited evidence in HNC, and existing studies are often constrained by small sample sizes due to the manual workflows [8, 23]. Automated delta biomarkers could offer a practical solution for integrating tumour dynamics into follow-up strategies, particularly in diseases like HNC where recurrence patterns are heterogeneous, and imaging is routinely used in surveillance [6, 7]. Structured imaging frameworks like Neck Imaging Reporting and Data System (NI-RADS) offer valuable guidance but do not incorporate personalised risk estimates based on quantitative tumour evolution [7].

There is therefore a gap in the current literature concerning the use of fully automated, AI-derived delta biomarkers for post-treatment risk stratification in HNC. To address this, we conducted an external validation study of a previously developed deep learning model for tumour segmentation on 18F-FDG PET/CT. Our primary aim was to evaluate whether deep learning-derived change in tumour volume (ΔPET-GTV) could stratify patients by loco-regional control following RT. A secondary aim was to evaluate whether the automatically generated segmentations were judged by local clinicians to be of sufficient quality to support GTV delineation, based on alignment with PET features and exclusion of non-malignant uptake. This was intended to inform the feasibility of clinical integration. The intended users of the model are oncology care teams seeking to personalise post-treatment surveillance intensity based on an individual estimate of risk of recurrence.

## Patients/material and methods

### Study design and setting

This was a retrospective external validation study conducted across two tertiary care centres. Model development was performed at Rigshospitalet (Copenhagen, Denmark), and external validation was conducted using patient data from The Christie NHS Foundation Trust (Manchester, UK). This study is reported in accordance with the Transparent Reporting of a multivariable prediction model for Individual Prognosis or Diagnosis – Artificial Intelligence (TRIPOD-AI) guideline [24]. In the external validation cohort, patients referred for RT with or without concomitant chemotherapy treated between February 2014 and September 2018 were included, with the last patient followed until July 2019. Details of the model development and initial validation cohorts are available in [25].

### Study population

Patients were retrospectively identified from the local research database (UK Computer-Aided Theragnostics (ukCAT) research database, Research Ethics Committee reference number: 21/NW/0347). Eligible patients had biopsy-confirmed HNC across multiple disease sites, including oropharynx, hypopharynx, nasopharynx, larynx and unknown primary (see Table 1) and underwent both pre- and post-treatment 18F-FDG PET/CT scans as part of routine care. All patients were treated at The Christie NHS Foundation Trust and received curative-intent RT either alone or in combination with chemotherapy, administered concurrently with cisplatin, carboplatin or cetuximab.

**Table 1:** Patient characteristics stratified by loco-regional failure (LRF) in the external validation cohort. Values are counts (percentages) for categorical variables or mean (standard deviation) for continuous variables. P-values indicate differences between LRF and non-LRF groups, calculated using chi-squared or Fisher’s exact test for categorical variables and ANOVA for continuous variables. Summary data from the training cohort are shown in the rightmost column for reference; limited clinical information was available for this cohort, as model development relied primarily on imaging and RT-struct DICOM files.

Variable	Overall (%)	No LRF (%)	LRF (%)	p	Training cohort
N	50	41	9		805
Sex = Male (%)	38 (76)	32 (78)	6 (66.7)	0.769	584 (73)
Age (mean, SD)	58.2 (7.7)	58.1 (7.9)	58.8 ().2	0.820	62.6
Site				0.028	
Hypopharynx	9 (18.0)	8 (19.5)	1 (11.1)		84 (10)
Larynx	4 (8.0)	3 (7.3)	1 (11.1)		123 (15)
Nasopharynx	7 (14.0)	7 (17.1)	0 (0.0)		50 (6)
Oropharynx	26 (52.0)	22 (52.7)	4 (44.4)		301 (37)
Unknown primary	4 (8.0)	1 (2.4)	3 (33.3)		18 (2)
Cavum oris, vesibulum nasi, sinus paranasalis, salivary gland tumour or unspecified	-	-	-		241 (30)
Histology				0.683	
Basaloid carcinoma	3 (6.0)	3 (7.3)	0 (0.0)		-
Other (non-kerat. NPC)¹	2 (4.0)	2 (4.9)	0 (0.0)		-
Other (susp. SCC)²	1 (2.0)	1 (2.5)	0 (0.0)		-
Other (SCC, NOS)³	44 (88.0)	35 (85.4)	9 (100.0)		-
p16 status				0.021	
Negative	8 (16.0)	4 (9.8)	4 (44.4)		-
Not applicable	20 (40.0)	18 (43.9)	2 (22.2)		-
Not known	5 (10.0)	3 (7.3)	2 (22.2)		-
Positive	17 (34.0)	16 (39.0)	1 (11.1)		-
ECOG performance status				0.682	
0	33 (66.0)	27 (65.9)	6 (66.7)		-
1	14 (28.0)	11 (26.8)	3 (33.3)		-
2	3 (6.0)	3 (7.3)	0 (0.0)		-
ACE-27 comorbidity score				0.260	
0	31 (62.0)	27 (65.9)	4 (44.4)		-
1	8 (16.0)	5 (12.2)	3 (33.3)		-
2	7 (14.0)	5 (12.2)	2 (22.2)		-
3	4 (8.0)	4 (9.8)	0 (0.0)		-
Smoking status				0.345	
Current smoker	22 (44.0)	16 (39.0)	6 (66.7)		-
Ex smoker	19 (38.0)	16 (39.0)	3 (33.3)		-
Life long never	8 (16.0)	8 (19.5)	0 (0.0)		-
Light former	1 (2.0)	1 (2.4)	0 (0.0)		-
T-stage				0.032	
1	9 (18.0)	8 (19.5)	1 (11.1)		-
2	16 (32.0)	14 (34.1)	2 (22.2)		-
3	5 (10.0)	5 (12.2)	0 (0.0)		-
4	16 (32.0)	13 (31.7)	3 (33.3)		-
X	4 (8.0)	1 (2.4)	3 (33.3)		-
N-stage				0.027	
0	2 (4.0)	2 (4.9)	0 (0.0)		-
1	5 (10.0)	5 (12.2)	0 (0.0)		-
2	36 (72.0)	31 (75.6)	5 (55.6)		-
3	7 (14.0)	3 (7.3)	4 (44.4)		-
CTV1 [cm³] (mean, SD)	188.76 (109.04)	176.81 (99.67)	243.22 (138.18)	0.098	-

**Notes**: ECOG: Eastern Cooperative Oncology Group performance status [31]. ACE-27: Adult Comorbidity Evaluation 27 [32].

¹ Other: non-keratinising undifferentiated nasopharyngeal carcinoma.

² Other: severe dysplasia suspicious of squamous cell carcinoma.

³ Other: squamous cell carcinoma, not otherwise specified (NOS).

### Image acquisition and pre-processing

All patients underwent a diagnostic 18F-FDG PET/CT scan before RT (pre-PET/CT), a planning CT scan for RT and a second PET/CT scan after RT (post-PET/CT), performed as part of routine clinical surveillance. Scans came from three PET/CT systems, and a patient’s two scans could be acquired on different machines. Clinical GTV definition was completed on the planning CT; physicians routinely reviewed the pre‑treatment PET/CT during GTV delineation.

PET images were resampled to the grid of their corresponding CT using linear interpolation; both modalities were cropped cranial-to-caudal to a fixed 35 cm axial extent, and DICOM files were converted to Nifti format. Preprocessing was applied uniformly across all patients for model training and evaluation.

Scanner models and acquisition protocols differed between the training and evaluation datasets. In the external validation cohort, 51 scans were acquired on a GE Discovery 710 system (slice thickness: 3.27 mm; pixel spacing: 3.65 mm), 45 on a GE Discovery STE (slice thickness: 3.27 mm; pixel spacing: 5.47 mm) and 4 on a Siemens Biograph 64 mCT (slice thickness: 3.00 mm; pixel spacing: 2.89 mm). In contrast, the training dataset consisted primarily of scans from Siemens Biograph 64 mCT and Vision systems with isotropic voxel spacing (~2.0 mm), and no GE scanners were included. Further details regarding the training dataset are available in [25].

Patient and acquisition characteristics were broadly comparable between cohorts: in the external validation cohort, mean weight, injected activity and uptake time were 72.4 ± 18.5 kg, 251.6 ± 66.3 MBq and 1.3 ± 0.2 h, respectively, compared to 75.0 ± 18.1 kg, 300.4 ± 72.1 MBq and 1.1 ± 0.2 h in the training cohort.

### AI model for PET-GTV segmentation

We applied a previously developed deep learning model for automated tumour segmentation on 18F-FDG PET/CT scans of HNC patients [25]. The model was trained on 805 pre-treatment scans using nnU-Net, which automatically configured an ensemble of low- and high-resolution 3D U-Net architectures and was selected as the best-performing framework amongst five tested approaches [17, 26–30]. Optimisation was based on a combined cross-entropy and Dice loss [17] with the Dice coefficient used as the primary evaluation metric to select the optimal model. In the present study, we evaluated the model outputs in a different context – using tumour volumes to stratify patients by LRF risk – to reflect a shift from technical to patient-relevant outcomes. The model was installed locally and applied without modification to the external PET/CT data. No re-training, fine-tuning or image registration was performed; images were processed directly by the model. Source code and model weights are available at https://github.com/CAAI/hnc-tumour-seg (further, see the section ‘Data Availability Statement’).

### Biomarker definition: ΔPET-GTV

To assess relative changes in tumour volume between baseline and follow-up scans, ΔPET-GTV was calculated as the relative difference: (pre − post)/pre. Patients were divided into two groups using the cohort median as a cut-off. Those with values above the median were assigned to the ‘High’ group (greater tumour shrinkage), and those below or equal to the median to the ‘Low’ group.

### Physician evaluation of segmentations

A qualitative assessment of AI-generated PET GTV segmentations on pre-treatment PET/CT-scans was performed by a single clinical oncology specialty trainee. Each case was reviewed using a structured questionnaire, focusing on clinical usability (Supplemental S1). The physician rated the segmentation quality on a four-point scale from poor to excellent, based on alignment with the PET gradient, exclusion of non-malignant uptake and overall suitability for guiding final GTV delineation. During the evaluation, the physician reviewed the AI-based PET-GTV volume overlaid on the pre-treatment PET/CT scan, with simultaneous access to the planning CT, including the clinically defined GTV, organs at risk and the associated RT dose distribution. This enabled simultaneous assessment of anatomical context and potential dosimetric impact in each case. Optional free-text fields were included to elaborate on individual responses. The qualitative evaluation did not involve post-treatment image assessment, allowing inclusion of all available pre-treatment PET/CT scans regardless of follow-up availability.

### Statistical analysis

The primary outcome was the probability of loco-regional control, derived using the time from baseline to loco-regional failure. Survival probabilities were estimated using Kaplan-Meier analysis, and differences between groups ‘Low’ and ‘High’ (see section ‘Biomarker Definition: ΔPET-GTV’) were assessed with the log-rank test. A sensitivity analysis excluding contours graded ‘Poor’ by the end‑user evaluator was performed to confirm the robustness of ΔPET‑GTV. To further quantify the association between tumour volume change and loco-regional control, a univariable Cox proportional hazards regression model was fitted. Model performance was assessed using the concordance index (C-index), and a model fit was evaluated using the likelihood ratio test. No formal sample size calculation was performed. The cohort included all eligible patients with complete imaging and outcome data during the study period. We acknowledge that the limited sample size reduces statistical power and increases the risk of Type II error. All analyses were conducted in R (version 4.3.1, 2023-06-16, supplemental S2–S6). No imputation was performed for missing data, and no sensitivity or secondary analyses were conducted. P-values of less than 0.05 were considered statistically significant.

## Results

### Patient characteristics

The validation cohort included 50 patients with HNC, pre- and post-PET/CT scans and follow-up information. Patient characteristics from the external validation and training cohort are included in Table 1 [31–32]. An additional 10 patients were included in the physician evaluation but were not part of the main study cohort due to missing outcome information (loco-regional failure status). The median age was 58 years (SD: 8), and the majority were male (76%). The most common primary tumour site was the oropharynx (52%), followed by the hypopharynx (18%) and nasopharynx (14%). Squamous cell carcinoma was the predominant histological subtype (88%).

Tumour stage was heterogeneous, with 32% of patients having T4 disease and 74% presenting with N2 nodal involvement. Regarding chemotherapy, 70% received cisplatin, 6% carboplatin and 2% cetuximab. Additionally, 4% were treated as part of the NIMRAD trial [33] with nimorazole, whilst 18% received RT alone.

**Figure 1:**
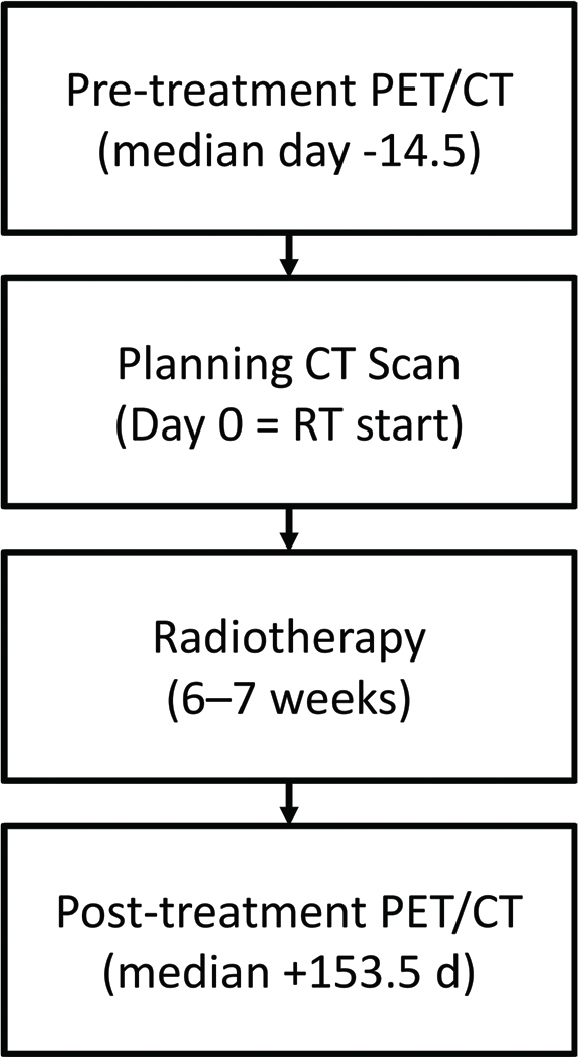
Overview of the imaging timeline relative to radiotherapy. All patients underwent a standard planning CT scan prior to treatment. In addition, a diagnostic 18F-FDG PET/CT scan was performed before radiotherapy (pre-PET/CT), and another PET/CT scan was acquired for response assessment after treatment (post-PET/CT). Median time from pre-treatment PET/CT to planning CT was 14.5 days (IQR: 0–31.8 days). Median time from pre- to post-treatment PET/CT was 153.5 days (IQR: 141.0–189.0 days).

Pre-treatment PET/CT scans were acquired a median of 14.5 days (interquartile range [IQR]: 0–31.75) before the planning CT, although in 12 cases, the PET/CT occurred after the planning CT (by up to 6 days). The median time from pre- to post-treatment PET/CT was 153.5 days (IQR: 141.0–189.0) (Figure 1). The median follow-up time was 360 days (SD: 286).

### Model performance and segmentation output

**Figure 2:**
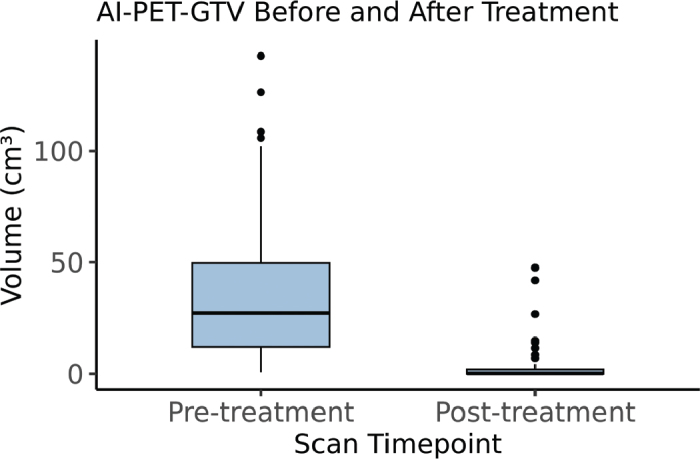
Boxplot showing the distribution of PET-GTV volumes from 18F-FDG PET/CT scans acquired before and after treatment. Each box represents the interquartile range (IQR), with the median indicated by the horizontal line. Volumes are shown in cubic centimetres (cm³). All scans were successfully segmented using the AI model.

All 100 scans (50 patients × 2 timepoints) were successfully processed using the deep learning segmentation model, with no failures due to missing data, artefacts or software errors. Whilst the quality of the segmentations varied (see ‘Physician evaluation’), no cases were excluded due to major segmentation errors such as inclusion of obvious physiological uptake or inflammatory response or omission of clearly malignant tissue. The automatic pre-treatment PET-GTV volumes had a median of 27.2 cm³ (IQR: 12.2–49.6 cm³), ranging from 0.7 to 142.6 cm³. Post-treatment volumes showed substantial reduction, with a median of 0.2 cm³ (IQR: 0–2.0 cm³) and a range from 0.0 to 47.6 cm³ (Figure 2). A paired t-test confirmed a significant mean decrease in tumour volume (p < 0.001).

### Delta biomarker distribution

The absolute difference in tumour volume had a mean of 33.7 cm³ (SD: 33.3 cm³), a median of 21.7 cm³ (IQR: 10.8–48.4 cm³) and ranged from –14.2 cm³ to 126.0 cm³. The relative difference had a mean of 0.494 (SD: 2.96), a median of 0.995 (IQR: 0.927–1.000) and ranged from –20.0 to 1.0. Negative values indicate tumour growth, whilst positive values correspond to tumour shrinkage.

### Loco-regional control outcomes

**Figure 3:**
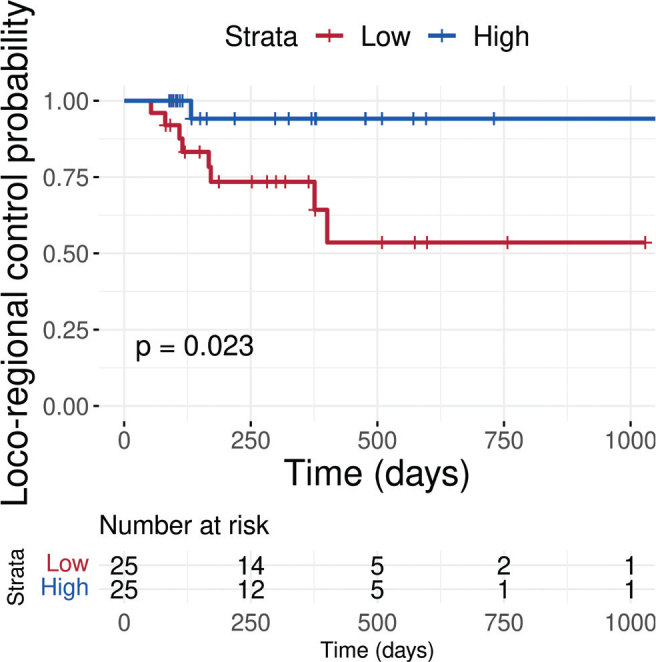
Kaplan-Meier curves showing time to loco-regional failure for patients stratified by relative tumour volume change. The group with lower relative shrinkage (‘Low’) had significantly poorer loco-regional control compared to the ‘High’ group (p = 0.02, log-rank test).

At 2 years, the estimated loco-regional control rate was 53.6% (95% CI: 33.2–89.1%) in the ‘Low’ group and 94.1% (95% CI: 83.6–100%) in the ‘High’ group (Figure 3). Loco-regional control differed significantly between the groups stratified by the median ΔPET-GTV (p = 0.02). Amongst patients in the ‘Low’ group (i.e. those with lower relative tumour shrinkage), 8 out of 25 experienced loco-regional failure, compared to only 1 out of 25 in the ‘High’ group. The univariable Cox proportional hazards model supported this finding, indicating a lower risk of loco-regional failure in the ‘High’ group, with an estimated hazard ratio of 0.13 (95% CI: 0.02–1.04, p = 0.055). Model concordance was 0.69, and the overall model fit was statistically significant according to the likelihood ratio test (p = 0.02). After excluding the 11 contours, the physician had rated ‘Poor’ during segmentation review, 39 of 50 patients remained (20 Low vs. 19 High ΔPET‑GTV). The log‑rank test for loco‑regional failure remained significant (p = 0.02), indicating that biomarker performance is unaffected by the removal of contours that the evaluator found sub-optimal for clinical use.

### Physician evaluation of segmentations

Of the 60 AI-generated segmentations evaluated, 47 (78.3%) were rated as acceptable or better (Figure S1). This included 17 segmentations (28.3%) rated as acceptable, 17 (28.3%) as good and 13 (21.7%) as excellent, whilst the remaining 13 segmentations (21.7%) were rated as poor. Figures S2 and S3 illustrate representative cases of AI-generated segmentations rated as acceptable and poor quality, respectively. In Figure S2, the evaluating physician noted that the difference between the GTV and AI-PET-GTV would have resulted in a larger primary tumour CTV and a shift of the high-dose region further into the trachea, leading to additional irradiation. In Figure S3, the segmentation was rated as poor due to omission of the base of tongue, where the tumour was located. Although clinically involved, this region lies near an air interface and often shows physiological FDG uptake, making its inclusion in PET-GTV challenging.

**Figure 4:**
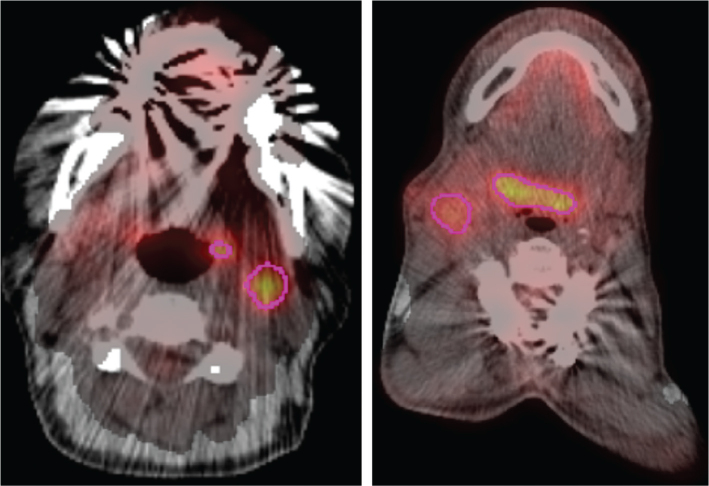
AI-PET-GTV output in two cases of unknown primary head and neck cancer. Left: A patient initially managed with surgical resection of a left-sided lymph node, later diagnosed with oropharyngeal cancer. The AI-PET-GTV correctly highlighted the oropharynx as the primary site on the pre-treatment PET-CT scan. Right: A patient with a right-sided nodal presentation who underwent nodal dissection before diagnosis of an oropharyngeal primary; here too, the AI-PET-GTV delineation correctly localised the oropharynx on the diagnostic scan. In both cases, the AI model identified the primary site before clinical diagnosis, supporting its potential value in unknown primary tumour localisation.

In two patients with unknown primary HNC, AI-PET-GTV correctly identified an oropharyngeal primary on the pre-treatment PET-CT scan, prior to clinical confirmation. In both cases, the AI delineation was considered appropriate and would not have altered nodal CTV but confirmed accurate localisation of the occult primary tumour (Figure 4).

## Discussion and conclusion

In this retrospective external validation study, we demonstrated that deep learning-derived change in PET-GTV (18F-FDG-avid part of gross tumour volume) can stratify patients by risk of loco-regional failure (LRF) following curative-intent RT for HNC. Patients with greater tumour shrinkage showed 2‑year control of 94% versus 54% in the low-shrinkage group. The algorithm ran fully automatically on routine PET/CTs, and in a separate secondary review, most pre‑treatment contours were judged clinically acceptable, supporting practical integration whilst highlighting the need for ongoing user‑centred evaluation.

Previous studies have shown that baseline or post-treatment PET parameters, such as SUVmax or metabolic tumour volume, correlate with treatment outcomes in HNC [4, 8, 9, 11]. However, these analyses have relied on manual segmentation, limiting clinical scalability. Delta biomarkers capturing serial changes in tumour characteristics have emerged as more predictive, with reviews in HNC demonstrating their potential value in outcome prediction [34, 35]. Our study builds on this by showing that a fully automated deep learning pipeline, applied externally and without manual intervention, can reproduce the known association between PET-derived tumour volume change and loco-regional control. This confirms that AI-derived ΔPET-GTV is both technically feasible and clinically meaningful for risk stratification in heterogeneous, real-world settings.

These findings support the potential utility of ΔPET-GTV for risk-adapted follow-up strategies in HNC. Current follow-up protocols often rely on fixed imaging schedules, which may lead to both under- and over-surveillance [6, 7]. A biomarker-based approach could enable more personalised surveillance aligned with individual recurrence risk. A key advantage of the algorithm is that it is fully deterministic, producing consistent segmentations for identical input data, which is particularly important when assessing longitudinal changes. Although ΔPET‑GTV is intended as a prognostic biomarker rather than a treatment‑planning contour, its eventual adoption still relies on clinicians understanding, and at least broadly agreeing with, the segmentations from which the metric is derived. In our external evaluation, 78% of contours were judged ‘acceptable’ or better. Even so, prognostic performance was unchanged when we re‑analysed the data after removing all cases whose segmentations the evaluator had labelled ‘Poor’, underscoring the biomarker’s robustness and the continuing need for user‑centred evaluation.

This study has several limitations. It was retrospective and included a relatively small number of cases. No image registration was applied between the PET and CT components of each scan, which may introduce geometric uncertainty from patient motion, though this also reflects a more efficient, registration-free workflow. The external validation site did not routinely delineate PET-GTVs as part of clinical practice, although physicians had access to pre-treatment PET/CT images during RT planning. Segmentation quality was assessed relative to the clinically defined GTV, which typically encompasses broader anatomical and clinical context. PET-GTV and GTV are conceptually different, with PET-GTV often representing a smaller subregion that contributes to, but does not define, the final GTV. The use of a less experienced evaluator may have influenced the ratings, as unfamiliarity with PET-GTV principles could lead to misinterpretation FDG-avid regions due to inflammatory response or physiological uptake. Further evaluation at centres where PET-GTV delineation is routinely performed is warranted. The median follow-up PET/CT interval (153.5 days) reflects typical 3–6-month surveillance; by this time, some loco-regional failures might already have been known, limiting the strictly prognostic interpretation of ΔPET-GTV. In such cases, the biomarker may act more as a response assessment tool than a predictive marker. Additionally, due to the skewed distribution of ΔPET-GTV values, dichotomisation may largely reflect the presence or absence of residual tumour. Moreover, the FDG-PET signal does not perfectly correspond to histological tumour extent and can be affected by post-treatment inflammation. However, one of the key strengths of the applied segmentation model is its ability to distinguish inflammatory from malignant uptake patterns, as previously demonstrated in qualitative examples [19]. Larger datasets are needed to refine cut-offs and explore alternative stratification strategies.

Although p16 status and initial tumour volume are established clinical predictors of loco-regional control, our aim was not to propose ΔPET-GTV as a replacement or superior alternative. Rather, we sought to validate whether our AI-based ΔPET-GTV-biomarker could produce clinically meaningful risk stratification in an independent, real-world cohort. Additional exploratory analyses did not find significant stratification by p16 status or initial tumour volume in this dataset (log-rank p = 0.1 for p16 and 1.0 for initial tumour volume), and multivariable modelling using p16 status and ΔPET-GTV as predictors suggested that ΔPET-GTV remained the most informative variable in this cohort (p = 0.058 for ΔPET-GTV vs. all p > 0.17 for p16); however, p16 status was missing or not applicable in 25 of 50 patients, limiting direct comparison. A focused analysis in Oropharyngeal squamous cell carcinoma (OPSCC)-only patients would be relevant, especially when aiming to show the independent predictive value of ΔPET-GTV but was not feasible here due to sample size and missing p16-status information. Meanwhile, for the purpose of this study, the fact that the model achieved stratification in this heterogeneous cohort supports its validity.

No fairness analysis across sociodemographic subgroups was performed due to limited sample size. Similarly, we did not assess heterogeneity in model performance across clinical subgroups such as tumour site or stage. No model updating or recalibration was performed, as the aim was to evaluate the model’s out-of-the-box performance on an independent dataset.

Key strengths of the study include independent external validation at a tertiary cancer centre, use of a previously trained model without fine-tuning and a fully automated inference pipeline that required no manual segmentation or image registration. The AI model used has been evaluated in multiple settings and is already approved for clinical use in some institutions [25]. Together, these features support its potential integration into scalable clinical workflows.

Recent frameworks such as LesionLocator [36] represent a promising direction in prompt-based, general-purpose lesion tracking across time and imaging modalities. In contrast, the model evaluated in our study is a specialised tool trained specifically for segmentation of PET-GTVs in head and neck 18F-FDG PET/CT and is integrated into a fully automated pipeline requiring no user prompts or interaction. Whilst prompt-based methods allow for flexible, user-guided lesion interpretation, our approach is designed for consistent, hands-free application to routine imaging, with outcome-based validation. A formal comparison of such generalised, interactive tools and task-specific, automated models would be valuable. As our model is openly available, it can be readily used as a benchmark for PET-GTV segmentation in future studies [19].

A logical next step following this study would be to evaluate the independent predictive value of ΔPET-GTV alongside other clinically relevant biomarkers, such as initial tumour volume, p16 status, smoking status and patient performance status. Such an analysis could help clarify the added prognostic value of ΔPET-GTV and determine, for example, whether it provides additional risk stratification in OPSCC patients treated with concomitant chemoradiotherapy. However, such analyses lie beyond the scope of the present work, which was specifically designed to test whether an AI-derived delta biomarker can enable meaningful post-treatment risk stratification in a clinically relevant, heterogeneous setting.

This study illustrates that fully automated, AI-derived delta PET biomarkers can quantify changes in tumour volume between pre- and post-treatment imaging and be used to stratify patients into high- and low-risk groups. Despite the absence of registration and physician input, the model was able to identify high-risk patients based on imaging data alone. Implementation depends on the availability of both pre- and post-treatment PET/CT scans; in cases where such imaging is unavailable, the model cannot be applied. Users are not required to interact with the segmentation process, although user expertise may be beneficial when reviewing the segmentation outputs that underlie specific predictions. In future implementations, anticipated barriers to clinical adoption will likely include differences in RT planning workflows across institutions. Additional challenges include the need for standardised validation frameworks and building clinician trust, echoing broader challenges described in the AI deployment literature [37]. Future implementation should include the development of a clinician-facing report interface that clearly communicates model outputs and segmentation reliability. However, this lies beyond the scope of the present work.

Deep learning-derived ΔPET-GTV offers a promising tool for assessing post-treatment disease status and supporting risk stratification in HNC. This study provides the first evidence of its external validity and supports its potential integration into personalised follow-up strategies. Broader validation and health-system integration are warranted.

## Supplementary Material



## Data Availability

The deep learning model and the training data used for this study are publicly available via the Rigshospitalet Tumour Segmentation website: https://rigshospitalet-tumour-segmentation.regionh.dk/. Due to the potential for patient re-identification from facial features in PET/CT imaging, the internal image data used in this study are not openly available. Access to these data may be granted upon reasonable request and following the signing of a data sharing agreement with Rigshospitalet. External validation image data were collected in the UK and cannot be shared publicly due to data protection regulations. Access requests require approval from relevant institutional data governance bodies.
