# Supplementary material for: External validation of deep learning-derived 18F-FDG PET/CT delta biomarkers for loco-regional control in head and neck cancer
Source: Acta Oncol. 2025 Aug 30;64:43977. doi: 10.2340/1651-226X.2025.43977 (PMC12406743; doi:10.2340/1651-226X.2025.43977)
Supplement: Supplementary file 1 [file AO-64-43977-s1.pdf]

Supplementary material has been published as submitted. It has not been copyedited, or typeset by Acta Oncologica

Supplemental material

For "External Validation of Deep Learning-Derived Δ18F-FDG PET/CT Biomarkers for Loco-Regional Control in Head and Neck Cancer: A Retrospective Risk Stratification Study".

Indhold

Supplemental material 1
S1 AI PET GTV evaluation – Questionnaire 2
S2_patient_characteristics 8
S3 Characterization of AI-PET-GTV 9
S4 Delta tumour volume distribution 11
S5 Kaplan Meier and Cox models 12
S6 Physician segmentation evaluation summary 14
S7 Supplementary Figures 18
S8 TRIPOD-AI Checklist 21

S1 AI PET GTV evaluation – Questionnaire

AI PET GTV Evaluation

As part of clinical routine at Rigshospitalet in Copenhagen, the PET GTV delineated by nuclear medicine specialists is sent to a radiation oncologist or radiologist, who uses the volume to guide the final GTV delineation. The PET GTV region was delineated on the PET image with an optionally underlying CT image. A visually adapted isocontour without a fixed threshold was used to fit the steepest gradient between the 18F-FDG–avid malignant region and the surrounding tissue, excluding areas with nonmalignant uptake.

AI PET GTV includes the primary tumour as well as malignant lymph nodes.

* Indicates required question

1. Enter your email *

   ____

2. Patient ukcat ID (Prefilled but still visible for confirmation - Required) *

   If you are testing, just add a random ID.

   ____

3. Pre-treatment PET-CT date (Prefilled but still visible for confirmation - Required) *

   ____

4. **Date of Planning CT** *(Date Picker, Required)* *

   ____

   Example: January 7, 2019

Volume quality assessment

📌 **Instruction:** *In this section, please evaluate the PET GTV volume itself, considering how well it aligns with the PET gradient, excludes nonmalignant uptake, and provides a useful basis for final GTV delineation.*

1️⃣ **Poor Quality** – The delineation is **not useful** for guiding final GTV delineation due to major errors, such as missing tumor regions, excessive inclusion of nonmalignant uptake, or poor alignment with expected PET/CT features.
2️⃣ **Acceptable Quality** – The delineation is **partially useful** but requires **significant adjustments** due to moderate over- or under-segmentation, or insufficient alignment with the steepest PET gradient.
3️⃣ **Good Quality** – The delineation is **mostly useful** and aligns well with the PET gradient and CT information, but **minor refinements** are needed to exclude nonmalignant uptake or adjust boundaries.
4️⃣ **Excellent Quality** – The delineation is **fully appropriate** for guiding final GTV delineation, accurately following the PET gradient while excluding nonmalignant uptake, requiring little or no modification.

5. **How would you rate the quality of the AI PET GTV delineation?** *(Likert Scale, Required)* *

Mark only one oval.

- ◯ Poor Quality (Not useful, major over/under-segmentation)
- ◯ Acceptable Quality (Partially useful, but needs significant adjustments)
- ◯ Good Quality (Mostly useful, minor refinements needed)
- ◯ Excellent Quality (Fully appropriate, minimal to no modification needed)

Impact on CTV – Primary Tumor

📌 **Instruction:** *In this section, please assess the AI PET GTV volume* ***relative to the CTV*** *and consider how following the AI PET GTV would have affected the final primary tumor CTV.*

6. **In what way would following the AI-PET-GTV have changed the assessment of the primary tumor CTV?** *(Multiple Choice, Required)* *

Mark only one oval.

- It would not change
- Would result in a larger CTV
- Would result in a smaller CTV
- Larger in some regions, smaller in others

7. Why? (Optional explanation)

Impact on CTV – Lymph Nodes

📌 **Instruction:** *Now, assess the AI PET GTV volume **relative to the CTV** for lymph nodes and consider how following the AI PET GTV would have affected the nodal CTV.*

8. **In what way would following the AI-PET-GTV have changed the assessment of the nodal CTV?** *(Multiple Choice, Required)* *

Mark only one oval.

- It would not change
- Would result in a larger CTV
- Would result in a smaller CTV
- Larger in some regeions, smaller in others

9. **Why? (Optional Explanation)** *(Short Answer, Optional)*

Overall Impact on Treatment Planning

📌 **Instruction:** *In this section, please consider the AI PET GTV, the final CTV, and the actual dose distribution. Assess how following the AI PET GTV would have influenced the irradiated area.*

10. **Would following the AI-PET-GTV have resulted in a different dose distribution?** *(Multiple Choice, Required)* *

Mark only one oval.

- No, the dose distribution would have remained the same
- Yes, it would have resulted in irradiating a larger area
- Yes, it would have resulted in irradiating a smaller area
- Yes, it would have resulted in irradiating a larger area in some regions and a smaller area in others

11. **Please elaborate on your evaluation of the potential dose distribution changes (if applicable).** *(Short Answer, Optional)*

This content is neither created nor endorsed by Google.

Google Forms

S2_patient_characteristics

```r
# Load required libraries
library(tidyverse)
library(readr)
library(tableone)

# File paths
input_path <- "path/to/data.csv"
output_csv <- "path/to/data.csv"

# Load dataset
df <- read_csv(input_path)

# Add ΔPET-GTV if not already calculated
df <- df %>%
  mutate(delta_tumor_volume = scan_1_x - scan_2_x)

# Convert LRF_status to factor
df <- df %>%
  mutate(LRF_status = factor(LRF_status, levels = c(0, 1), labels = c("No LRF",
"LRF")))

# Define variables to summarise
vars <- c("sex", "age", "site", "histology", "ecog_ps", "ACE27_overall",
          "smoking_status", "T_stage", "N_stage", "CTV1",
          "scan_1_x", "scan_2_x", "delta_tumor_volume")

# Create stratified Table 1 with overall column
table1 <- CreateTableOne(vars = vars, data = df, strata = "LRF_status",
                         factorVars = c("sex", "site", "histology",
                                        "ecog_ps", "ACE27_overall",
                                        "smoking_status", "T_stage", "N_stage"),
                         addOverall = TRUE)

# Convert to data frame for export
table1_df <- print(table1, quote = FALSE, noSpaces = TRUE, printToggle = FALSE)

# Add rownames as a column
table1_df <- tibble::rownames_to_column(as.data.frame(table1_df), var = "Variable")

# Indent subcategories for grouped variables
# We'll detect these as entries where the Variable name starts with whitespace or
special characters (like "  " or numbers)
indent_subrows <- function(var_names) {
```

```
  var_names <- gsub("\\.+", " ", var_names)                          # Replace ..
and ... with space
  var_names <- gsub("\\.\\d+$", "", var_names)                       # Remove
suffixes like ".1", ".2"
  var_names <- gsub("^\\s+|\\s+$", "", var_names)                    # Trim
  var_names <- ifelse(grepl("^[0-9]|^Current|^Ex
|^Life|^Light|^Basaloid|^Other|^Squamous|^hypo|^larynx|^naso|^oro|^unknown|^X|^1
|^2 |^3", var_names),
                      paste0("  ", var_names), var_names)           # Add two
spaces for subcategories
  return(var_names)
}

table1_df$Variable <- indent_subrows(table1_df$Variable)

# Save cleaned table to CSV
write_csv(table1_df, output_csv)
```

S3 Characterization of AI-PET-GTV

```
# Load required libraries
library(tidyverse)
library(ggplot2)

# Load the dataset
df <- read_csv("/mnt2/ext_valid/clinical_factors_with_tvols_suv_gtv_2.csv")

# Summarise pre- and post-treatment PET-GTV volumes
summary_stats <- df %>%
  summarise(
    n = n(),
    pre_median = median(scan_1_x, na.rm = TRUE),
    pre_iqr = IQR(scan_1_x, na.rm = TRUE),
    pre_mean = mean(scan_1_x, na.rm = TRUE),
    pre_sd = sd(scan_1_x, na.rm = TRUE),
    pre_min = min(scan_1_x, na.rm = TRUE),
    pre_max = max(scan_1_x, na.rm = TRUE),

    post_median = median(scan_2_x, na.rm = TRUE),
    post_iqr = IQR(scan_2_x, na.rm = TRUE),
    post_mean = mean(scan_2_x, na.rm = TRUE),
    post_sd = sd(scan_2_x, na.rm = TRUE),
    post_min = min(scan_2_x, na.rm = TRUE),
    post_max = max(scan_2_x, na.rm = TRUE)
  )
```

```
# Print the result

summary(df$scan_1_x)
summary(df$scan_2_x)
1
# Perform a paired t-test
t_test_result <- t.test(df$scan_1_x, df$scan_2_x, paired = TRUE)

# Print the t-test result
print(t_test_result)

# plots
# Prepare data for boxplot
df_long <- df %>%
  pivot_longer(
    cols = c(scan_1_x, scan_2_x),
    names_to = "ScanTime",
    values_to = "Volume_cm3"
  ) %>%
  mutate(
    ScanTime = recode(
      ScanTime,
      scan_1_x = "Pre-treatment",
      scan_2_x = "Post-treatment"
    ),
    # Set the order of the factor levels
    ScanTime = factor(ScanTime, levels = c("Pre-treatment", "Post-treatment"))
  )

# Create boxplot
ggplot(df_long, aes(x = ScanTime, y = Volume_cm3)) +
  geom_boxplot(fill = "#A3C1DA", colour = "black", width = 0.5) +
  labs(
    title = "AI-PET-GTV Tumour Volumes Before and After Treatment",
    x = "Scan Timepoint",
    y = "Volume (cm³)"
  ) +
  theme_classic(base_size = 12) +
  theme(
    axis.text.x = element_text(size = 12),  # Increase x-axis tick size
    axis.text.y = element_text(size = 12),
    plot.title = element_text(size = 12)   # Decrease title size
  )

# Save the plot
```

```r
ggsave("/mnt2/ext_valid/analysis_output/boxplot_tumour_volumes.png", width = 6,
height = 4, dpi = 300)

df_long$ScanTime <- factor(df_long$ScanTime, levels = c("Pre-treatment", "Post-
treatment"), labels = c("Pre", "Post"))


# Create simplified boxplot for graphical abstract
ggplot(df_long, aes(x = ScanTime, y = Volume_cm3)) +
  geom_boxplot(fill = "#A3C1DA", colour = "black", width = 0.5) +
  labs(
    x = "",
    y = "Tumour volume (cm³)"
  ) +
  theme_classic(base_size = 16) +
  theme(
    axis.text.x = element_text(size = 16),
    axis.text.y = element_text(size = 16),
    plot.title = element_blank()
  )

# Save the plot
ggsave("/mnt2/ext_valid/analysis_output/boxplot_tumour_volumes-icon.png", width =
4, height = 4, dpi = 300)
```

S4 Delta tumour volume distribution

```r
# Load required libraries
library(tidyverse)
library(readr)
library(ggplot2)

# File path
input_path <- "/mnt2/ext_valid/clinical_factors_with_tvols_suv_gtv_2.csv"

# Load dataset
df <- read_csv(input_path)

# Summary statistics
summary_stats <- df %>%
  summarise(
    n = n(),
    mean = mean(delta_tumor_volume),
    sd = sd(delta_tumor_volume),
```

```r
    median = median(delta_tumor_volume),
    iqr = IQR(delta_tumor_volume),
    min = min(delta_tumor_volume),
    max = max(delta_tumor_volume)
  )
print(summary_stats)

# Dichotomisation by median
median_threshold <- median(df$delta_tumor_volume, na.rm = TRUE)
df <- df %>%
  mutate(delta_group = ifelse(delta_tumor_volume > median_threshold, "Above
median", "Below or equal to median"))

# Count in each group
group_counts <- df %>%
  count(delta_group)
print(group_counts)


print(sum(df$delta_tumor_volume < 0, na.rm = TRUE))
```

S5 Kaplan Meier and Cox models

```r
rm(list=ls())

# Load required libraries
library(survival)         # For Cox regression & Kaplan-Meier analysis
library(survminer)        # For Kaplan-Meier visualization
library(dplyr)            # For data manipulation
library(riskRegression)  # For Score() function

# Define file paths
merged_dataset_path <- "/my/path/clinical_factors_with_tvols_suv_gtv_2.csv"
output_analysis_folder <- "/my/path/analysis_output"
output_model_summary_path <- file.path(output_analysis_folder,
"cox_model_summaries.txt")
output_log_rank_path <- file.path(output_analysis_folder, "log_rank_tests.txt")
output_km_plot_path <- file.path(output_analysis_folder, "KM_plots.png")
output_km_plot_path_delta_pet_gtv <- file.path(output_analysis_folder,
"KM_plot_delta_pet_gtv.png")
output_roc_plot_path_delta_pet_gtv <- file.path(output_analysis_folder,
"roc_delta_pet_gtv.png")


# Load the dataset
```

```r
data <- read.csv(merged_dataset_path)
data$scan_1_suv_max <- data$scan_1_y
data$scan_2_suv_max <- data$scan_2_y
data$delta_suv_max <- data$delta_suv_max

# Define small pseudo-count for log transformation
epsilon <- 0.01

# Compute log-transformed tumor volumes
data <- data %>%
  mutate(
    sex_binary = ifelse(sex == "Male", 1, 0),
    log_scan_1_tvol = log(scan_1_tvol),
    log_scan_2_tvol = log(scan_2_tvol),
    Δ_log_tvol = log((scan_1_tvol + epsilon) / (scan_2_tvol + epsilon))
    )


# Define median cutoffs for stratifying groups
median_Δ_AI_PET_GTV <- median(data$Δ_log_tvol, na.rm = TRUE)
median_Δ_AI_PET_GTV

# Categorize groups based on median values (LOW as reference category)
data <- data %>%
  mutate(
    group_Δ_AI_GTV = factor(ifelse(Δ_log_tvol > median_Δ_AI_PET_GTV, "High",
"Low"), levels = c("Low", "High")),
  )

# Define Cox models
cox_AI_delta_vol <- coxph(Surv(time_to_LRF_days, LRF_status) ~ group_Δ_AI_GTV, data
= data)

# Save Cox model summaries to a text file
writeLines(capture.output({
  cat("\nCox Model (AI Predictors - delta - Tvol)\n")
  print(summary(cox_AI_delta_vol))
}), con = output_model_summary_path)

# Define custom colors (matching your preference)
custom_colors <- c("#B30838", "#0858B3")  # Red for High, Blue for Low

# Define Kaplan-Meier survival objects
km_AI_PET_GTV <- survfit(Surv(time_to_LRF_days, LRF_status) ~ group_Δ_AI_GTV, data
= data)

summary(km_AI_PET_GTV, times = c(2*365.25))
```

```
# Perform log-rank test for significance
log_rank_tests <- list(
  AI_PET_GTV = survdiff(Surv(time_to_LRF_days, LRF_status) ~ group_Δ_AI_GTV, data =
data)
)

# Save log-rank test results to a text file
writeLines(capture.output({
  cat("### Log-Rank Test Results ###\n\n")
  for (test in names(log_rank_tests)) {
    cat("Log-Rank Test for", test, "\n")
    print(log_rank_tests[[test]])
    cat("\n--------------------------------------\n")
  }
}), con = output_log_rank_path)



# Save Kaplan-Meier plots to PNG file
png(output_km_plot_path_delta_pet_gtv, width = 1600, height = 1600, res = 300)

# Generate Kaplan-Meier plots with larger text sizes
km_plot1 <- ggsurvplot(km_AI_PET_GTV, data = data, risk.table = TRUE, pval = TRUE,
                        ggtheme = theme_minimal(),
                        legend.labs = c("Low", "High"),
                        palette = custom_colors,
                        xlab = "Time (days)", ylab = "Loco-regional control
probability",
                        font.title = 20, font.legend = 14, font.x = 18, font.y =
18)

# Arrange plots in a 2x2 grid
km_plot1
# Close the graphics device to save the file
dev.off()

cat("Kaplan-Meier plot saved to:", output_km_plot_path_delta_pet_gtv, "\n")
cat("Cox model summaries saved to:", output_model_summary_path, "\n")
cat("Log-rank test results saved to:", output_log_rank_path, "\n")
```

S6 Physician segmentation evaluation summary

```
rm(list=ls())
```

```r
# Load required packages
library(ggplot2)
library(gridExtra)

# Read the data
data <- read.csv("AI PET GTV evaluation (Responses) - Form Responses 1.csv",
stringsAsFactors = FALSE)

names(data) <- c("timestamp", "patient_id", "pet_ct_date", "planning_ct_date",
"quality_rating",
                 "primary_ctv_impact", "primary_ctv_explanation",
"nodal_ctv_impact", "nodal_ctv_explanation",
                 "dose_impact", "dose_explanation", "email_1", "email_2")


# Clean and relabel quality ratings
data$quality <- factor(data$quality_rating,
                       levels = c("Poor Quality (Not useful, major over/under-
segmentation)",
                                  "Acceptable Quality (Partially useful, but needs
significant adjustments)",
                                  "Good Quality (Mostly useful, minor refinements
needed)",
                                  "Excellent Quality (Fully appropriate, minimal to
no modification needed)"),
                       labels = c("Poor", "Acceptable", "Good", "Excellent"))

# Shorter labels for primary CTV impact
data$primary_ctv <- factor(data$primary_ctv_impact,
                           levels = c("It would not change",
                                      "Would result in a larger CTV",
                                      "Would result in a smaller CTV",
                                      "Larger in some regions, smaller in others"),
                           labels = c("No change", "Larger", "Smaller", "Mixed"))

# Shorter labels for nodal CTV impact
data$nodal_ctv <- factor(data$nodal_ctv_impact,
                         levels = c("It would not change",
                                    "Would result in a larger CTV",
                                    "Would result in a smaller CTV",
                                    "Larger in some regeions, smaller in
others"),  # Note the typo in "regeions"
                         labels = c("No change", "Larger", "Smaller", "Mixed"))

# Shorter labels for dose distribution impact
data$dose <- factor(data$dose_impact,
```

```
                    levels = c("No, the dose distribution would have remained the 
same",
                               "Yes, it would have resulted in irradiating a larger 
area",
                               "Yes, it would have resulted in irradiating a 
smaller area",
                               "Yes, it would have resulted in irradiating a larger 
area in some regions and a smaller area in others"),
                    labels = c("No change", "Larger", "Smaller", "Mixed"))

# Absolute and relative frequencies
# Segmentation quality
cat("Segmentation Quality Ratings (absolute):\n")
print(table(data$quality))

cat("\nSegmentation Quality Ratings (relative %):\n")
print(round(100 * prop.table(table(data$quality)), 1))

# Primary CTV impact
cat("\nImpact on Primary Tumor CTV (absolute):\n")
print(table(data$primary_ctv))

cat("\nImpact on Primary Tumor CTV (relative %):\n")
print(round(100 * prop.table(table(data$primary_ctv)), 1))

# Nodal CTV impact
cat("\nImpact on Nodal CTV (absolute):\n")
print(table(data$nodal_ctv))

cat("\nImpact on Nodal CTV (relative %):\n")
print(round(100 * prop.table(table(data$nodal_ctv)), 1))

# Dose distribution
cat("\nExpected Dose Distribution Change (absolute):\n")
print(table(data$dose))

cat("\nExpected Dose Distribution Change (relative %):\n")
print(round(100 * prop.table(table(data$dose)), 1))

# Create output directory if it doesn't exist
output_dir <- "/mnt2/ext_valid/analysis_output"

# Plot 1: Segmentation Quality
p1 <- ggplot(data, aes(x = quality)) +
  geom_bar(fill = "steelblue") +
  theme_minimal() +
  ylab("Number of Cases") +
```

```r
  xlab("Segmentation Quality Rating") +
  ggtitle("AI PET GTV Quality")+
  theme(
    axis.text = element_text(size = 12),
    axis.title = element_text(size = 14),
    plot.title = element_text(size = 16, face = "bold")
  )
ggsave(file.path(output_dir, "qualitative_segmentation_quality.png"), p1, width =
6, height = 4, dpi = 300)

# Plot 2: Primary CTV Impact
p2 <- ggplot(data, aes(x = primary_ctv)) +
  geom_bar(fill = "darkgreen") +
  theme_minimal() +
  ylab("Number of Cases") +
  xlab("Impact on Primary Tumor CTV") +
  ggtitle("Segmentation on Primary Tumor CTV") +
  theme(
    axis.text = element_text(size = 12),
    axis.title = element_text(size = 14),
    plot.title = element_text(size = 16, face = "bold")
  )
ggsave(file.path(output_dir, "qualitative_primary_ctv_impact.png"), p2, width = 6,
height = 4, dpi = 300)

# Plot 3: Nodal CTV Impact
p3 <- ggplot(data, aes(x = nodal_ctv)) +
  geom_bar(fill = "purple") +
  theme_minimal() +
  ylab("Number of Cases") +
  xlab("Impact on Nodal CTV") +
  ggtitle("Segmentation on Nodal CTV") +
  theme(
    axis.text = element_text(size = 12),
    axis.title = element_text(size = 14),
    plot.title = element_text(size = 16, face = "bold")
  )
ggsave(file.path(output_dir, "qualitative_nodal_ctv_impact.png"), p3, width = 6,
height = 4, dpi = 300)

# Plot 4: Dose Distribution Impact
p4 <- ggplot(data, aes(x = dose)) +
  geom_bar(fill = "orange") +
  theme_minimal() +
  ylab("Number of Cases") +
  xlab("Expected Dose Distribution Change") +
  ggtitle("Segmentation on Dose Distribution") +
```

```r
  theme(
    axis.text = element_text(size = 12),
    axis.title = element_text(size = 14),
    plot.title = element_text(size = 16, face = "bold")
  )
ggsave(file.path(output_dir, "qualitative_dose_distribution_impact.png"), p4, width
= 6, height = 4, dpi = 300)

# Combine all 4 plots into one figure
combined_plot <- grid.arrange(p1, p2, p3, p4, ncol = 2)
ggsave(file.path(output_dir, "qualitative_combined_summary_plots.png"),
combined_plot, width = 12, height = 8, dpi = 300)
```

S7 Supplementary Figures

Figure S1: Physician evaluation of AI-generated PET GTV segmentations (n = 60).

Bar plot summarising physicians' qualitative ratings of segmentation quality for AI-PET-GTV. A majority of segmentations (78.3%) were rated as acceptable or better.

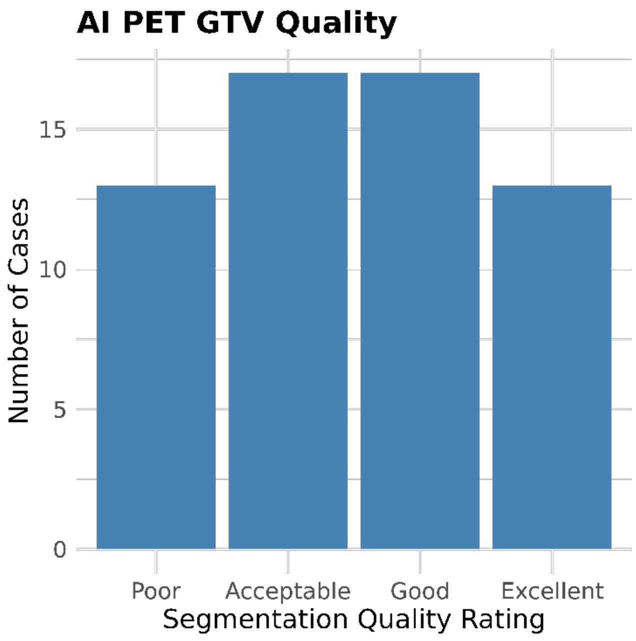

Figure S2: Example case with an AI-generated PET GTV rated as Acceptable Quality.

The top panel shows the pre-treatment PET-CT with the AI-PET-GTV overlaid in cyan. The bottom panel shows the planning CT with the clinically defined GTV in red and the CTV in blue. The planning CT was acquired 3 days after the pre-treatment PET-CT. This patient was diagnosed with T2N0 laryngeal squamous cell carcinoma. The AI-generated PET GTV was rated as acceptable quality by the physician. Compared to the clinically defined GTV, the AI-PET-GTV extended further inferiorly, including the lower larynx and part of the upper trachea.

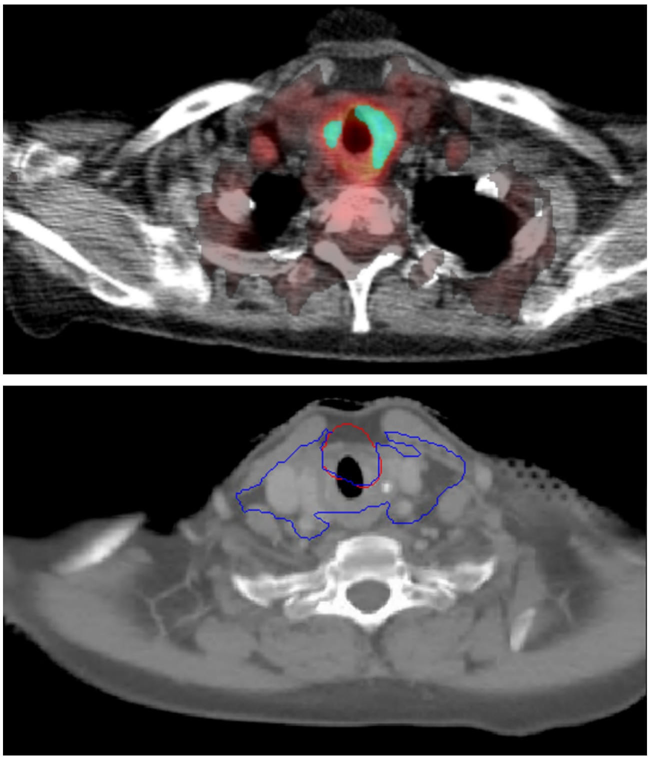

Figure S3: Example case with an AI-generated PET GTV rated as Poor Quality.

The left panel shows the PET-CT with the AI-PET-GTV overlaid in cyan. The right panel shows the planning CT with clinical target volumes: orange for CTVp (primary tumour), red for CTV (nodes), and blue for the elective volume. This patient was diagnosed with T2N2 hypopharyngeal squamous cell carcinoma. The AI-PET-GTV was rated as poor quality by the physician.

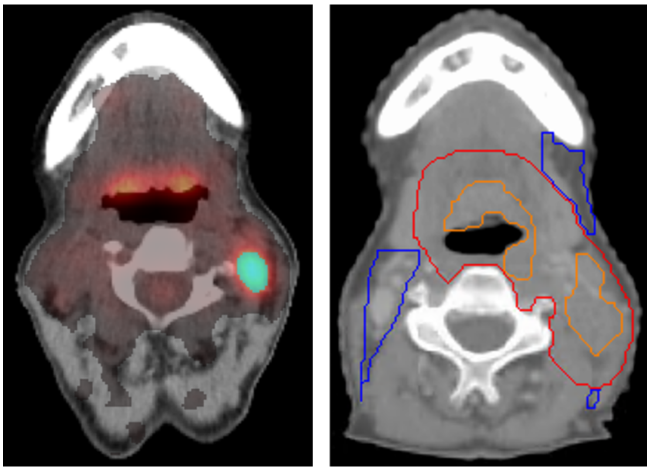

S8 TRIPOD-AI Checklist

TRIPOD+AI

Version: 11-January-2024

Section/Topic	Item	Development / evaluation[1]	Checklist item	Reported on page
**TITLE**				
Title	1	D;E	Identify the study as developing or evaluating the performance of a multivariable prediction model, the target population, and the outcome to be predicted	1
**ABSTRACT**				
Abstract	2	D;E	See TRIPOD+AI for Abstracts checklist	3
**INTRODUCTION**				
Background	3a	D;E	Explain the healthcare context (including whether diagnostic or prognostic) and rationale for developing or evaluating the prediction model, including references to existing models	4
	3b	D;E	Describe the target population and the intended purpose of the prediction model in the context of the care pathway, including its intended users (e.g., healthcare professionals, patients, public)	4
	3c	D;E	Describe any known health inequalities between sociodemographic groups	4
Objectives	4	D;E	Specify the study objectives, including whether the study describes the development or validation of a prediction model (or both)	5
**METHODS**				
Data	5a	D;E	Describe the sources of data separately for the development and evaluation datasets (e.g., randomised trial, cohort, routine care or registry data), the rationale for using these data, and representativeness of the data	5
	5b	D;E	Specify the dates of the collected participant data, including start and end of participant accrual; and, if applicable, end of follow-up	5
Participants	6a	D;E	Specify key elements of the study setting (e.g., primary care, secondary care, general population) including the number and location of centres	5
	6b	D;E	Describe the eligibility criteria for study participants	TBD
	6c	D;E	Give details of any treatments received, and how they were handled during model development or evaluation, if relevant	5
Data preparation	7	D;E	Describe any data pre-processing and quality checking, including whether this was similar across relevant sociodemographic groups	5
Outcome	8a	D;E	Clearly define the outcome that is being predicted and the time horizon, including how and when assessed, the rationale for choosing this outcome, and whether the method of outcome assessment is consistent across sociodemographic groups	6
	8b	D;E	If outcome assessment requires subjective interpretation, describe the qualifications and demographic characteristics of the outcome assessors	NA
	8c	D;E	Report any actions to blind assessment of the outcome to be predicted	NA
Predictors	9a	D	Describe the choice of initial predictors (e.g., literature, previous models, all available predictors) and any pre-selection of predictors before model building	6
	9b	D;E	Clearly define all predictors, including how and when they were measured (and any actions to blind assessment of predictors for the outcome and other predictors)	6
	9c	D;E	If predictor measurement requires subjective interpretation, describe the qualifications and demographic characteristics of the predictor assessors	NA
Sample size	10	D;E	Explain how the study size was arrived at (separately for development and evaluation), and justify that the study size was sufficient to answer the research question. Include details of any sample size calculation	7
Missing data	11	D;E	Describe how missing data were handled. Provide reasons for omitting any data	5
Analytical methods	12a	D	Describe how the data were used (e.g., for development and evaluation of model performance) in the analysis, including whether the data were partitioned, considering any sample size requirements	NA
	12b	D	Depending on the type of model, describe how predictors were handled in the analyses (functional form, rescaling, transformation, or any standardisation).	NA
	12c	D	Specify the type of model, rationale[2], all model-building steps, including any hyperparameter tuning, and method for internal validation	NA
	12d	D;E	Describe if and how any heterogeneity in estimates of model parameter values and model performance was handled and quantified across clusters (e.g., hospitals, countries). See TRIPOD-Cluster for additional considerations[3]	NA (single site)
	12e	D;E	Specify all measures and plots used (and their rationale) to evaluate model performance (e.g., discrimination, calibration, clinical utility) and, if relevant, to compare multiple models	7
	12f	E	Describe any model updating (e.g., recalibration) arising from the model evaluation, either overall or for particular sociodemographic groups or settings	NA
	12g	E	For model evaluation, describe how the model predictions were calculated (e.g., formula, code, object, application programming interface)	6
Class imbalance	13	D;E	If class imbalance methods were used, state why and how this was done, and any subsequent methods to recalibrate the model or the model predictions	NA
Fairness	14	D;E	Describe any approaches that were used to address model fairness and their rationale	9
Model output	15	D	Specify the output of the prediction model (e.g., probabilities, classification). Provide details and rationale for any classification and how the thresholds were identified	7

[1] D=items relevant only to the development of a prediction model; E=items relating solely to the evaluation of a prediction model; D;E=items applicable to both the development and evaluation of a prediction model

[2] Separately for all model building approaches.

[3] TRIPOD-Cluster is a checklist of reporting recommendations for studies developing or validating models that explicitly account for clustering or explore heterogeneity in model performance (eg, at different hospitals or centres). Debray et al, BMJ 2023; 380: e071018 [DOI: 10.1136/bmj-2022-071018]

Training versus evaluation	16	D;E	Identify any differences between the development and evaluation data in healthcare setting, eligibility criteria, outcome, and predictors	6,7
Ethical approval	17	D;E	Name the institutional research board or ethics committee that approved the study and describe the participant-informed consent or the ethics committee waiver of informed consent	12
**OPEN SCIENCE**				
Funding	18a	D;E	Give the source of funding and the role of the funders for the present study	11
Conflicts of interest	18b	D;E	Declare any conflicts of interest and financial disclosures for all authors	11
Protocol	18c	D;E	Indicate where the study protocol can be accessed or state that a protocol was not prepared	12
Registration	18d	D;E	Provide registration information for the study, including register name and registration number, or state that the study was not registered	12
Data sharing	18e	D;E	Provide details of the availability of the study data	12
Code sharing	18f	D;E	Provide details of the availability of the analytical code[4]	7, 12
**PATIENT & PUBLIC INVOLVEMENT**				
Patient & Public Involvement	19	D;E	Provide details of any patient and public involvement during the design, conduct, reporting, interpretation, or dissemination of the study or state no involvement.	12
**RESULTS**				
Participants	20a	D;E	Describe the flow of participants through the study, including the number of participants with and without the outcome and, if applicable, a summary of the follow-up time. A diagram may be helpful.	8
	20b	D;E	Report the characteristics overall and, where applicable, for each data source or setting, including the key dates, key predictors (including demographics), treatments received, sample size, number of outcome events, follow-up time, and amount of missing data. A table may be helpful. Report any differences across key demographic groups.	8
	20c	E	For model evaluation, show a comparison with the development data of the distribution of important predictors (demographics, predictors, and outcome).	8
Model development	21	D;E	Specify the number of participants and outcome events in each analysis (e.g., for model development, hyperparameter tuning, model evaluation)	8
Model specification	22	D	Provide details of the full prediction model (e.g., formula, code, object, application programming interface) to allow predictions in new individuals and to enable third-party evaluation and implementation, including any restrictions to access or re-use (e.g., freely available, proprietary)[5]	7
Model performance	23a	D;E	Report model performance estimates with confidence intervals, including for any key subgroups (e.g., sociodemographic). Consider plots to aid presentation.	9
	23b	D;E	If examined, report results of any heterogeneity in model performance across clusters. See TRIPOD Cluster for additional details[3].	10
Model updating	24	E	Report the results from any model updating, including the updated model and subsequent performance	10
**DISCUSSION**				
Interpretation	25	D;E	Give an overall interpretation of the main results, including issues of fairness in the context of the objectives and previous studies	10
Limitations	26	D;E	Discuss any limitations of the study (such as a non-representative sample, sample size, overfitting, missing data) and their effects on any biases, statistical uncertainty, and generalizability	10
Usability of the model in the context of current care	27a	D	Describe how poor quality or unavailable input data (e.g., predictor values) should be assessed and handled when implementing the prediction model	11
	27b	D	Specify whether users will be required to interact in the handling of the input data or use of the model, and what level of expertise is required of users	11
	27c	D;E	Discuss any next steps for future research, with a specific view to applicability and generalizability of the model	11

From: Collins GS, Moons KGM, Dhiman P, et al. *BMJ* 2024;385:e078378. doi:10.1136/bmj-2023-078378

[4] This relates to the analysis code, for example, any data cleaning, feature engineering, model building, evaluation.
[5] This relates to the code to implement the model to get estimates of risk for a new individual.
